# Supplementary material for: AREG Upregulation in Cancer Cells via Direct Interaction with Cancer-Associated Fibroblasts Promotes Esophageal Squamous Cell Carcinoma Progression Through EGFR-Erk/p38 MAPK Signaling
Source: Cells. 2024 Oct 19;13(20):1733. doi: 10.3390/cells13201733 (PMC11506648; doi:10.3390/cells13201733)
Supplement: Supplementary file 1 [file cells-13-01733-s001.zip › cells-3194949-supplementary.pdf]

Supplementary Materials

AREG Upregulation in Cancer Cells via Direct Interaction with Cancer-Associated Fibroblasts Promotes Esophageal Squamous Cell Carcinoma Progression Through EGFR-Erk/p38 MAPK Signaling

Takashi Nakanishi, Yu-ichiro Koma, Shoji Miyako, Rikuya Torigoe, Hiroki Yokoo, Masaki Omori, Keitaro Yamanaka, Nobuaki Ishihara, Shuichi Tsukamoto, Takayuki Kodama, Mari Nishio, Manabu Shigeoka, Hiroshi Yokozaki, Yoshihiro Kakeji

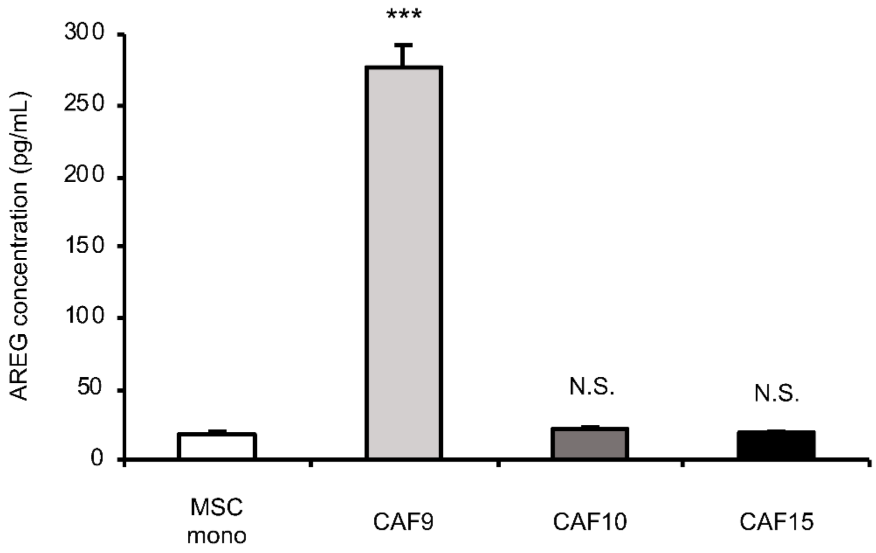

Figure S1. The secreted protein levels of AREG in MSC mono and CAFs were compared using enzyme-linked immunosorbent assay. N.S., not significant; *** $p < 0.001$.

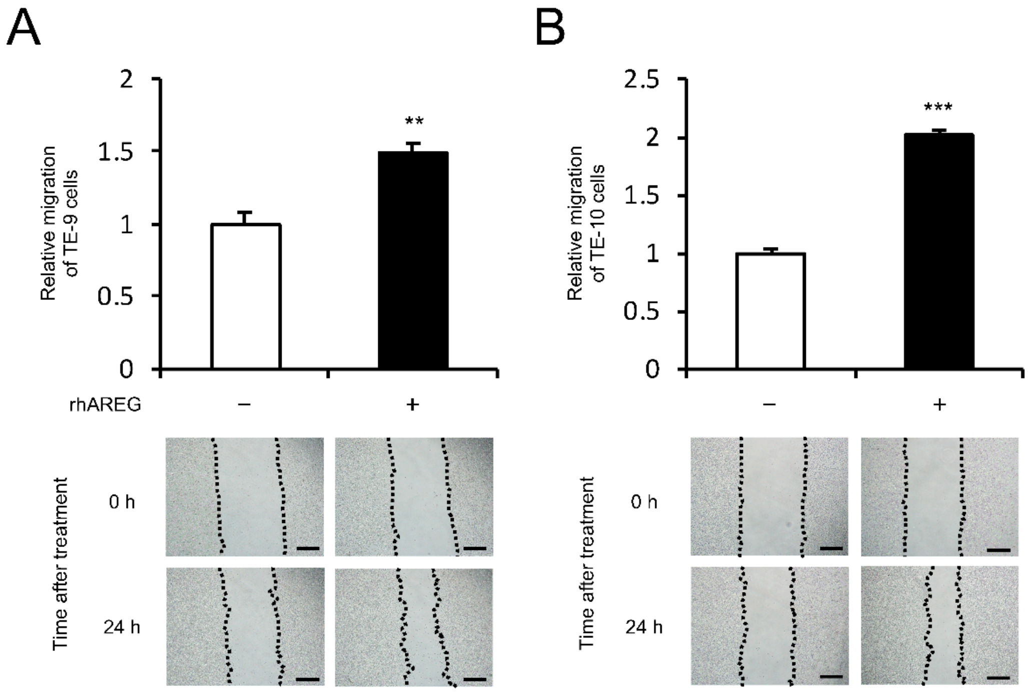

Figure S2. Wound healing assay showing the effect of recombinant human amphiregulin (rhAREG) (100 ng/mL) on the horizontal migration of esophageal squamous cell carcinoma (ESCC) cells. The wound closure rates of TE-9 **(A)** and TE-10 **(B)** were compared after 24 h of culture with or without rhAREG, following the creation of scratch wounds. Representative images for each condition are presented. The dotted lines in each image represent the scratch wounds. The graphs are presented as the mean ± standard error of the mean (SEM) of three independent experiments. ** $p < 0.01$, *** $p < 0.001$. Scale bars: 100 μm.

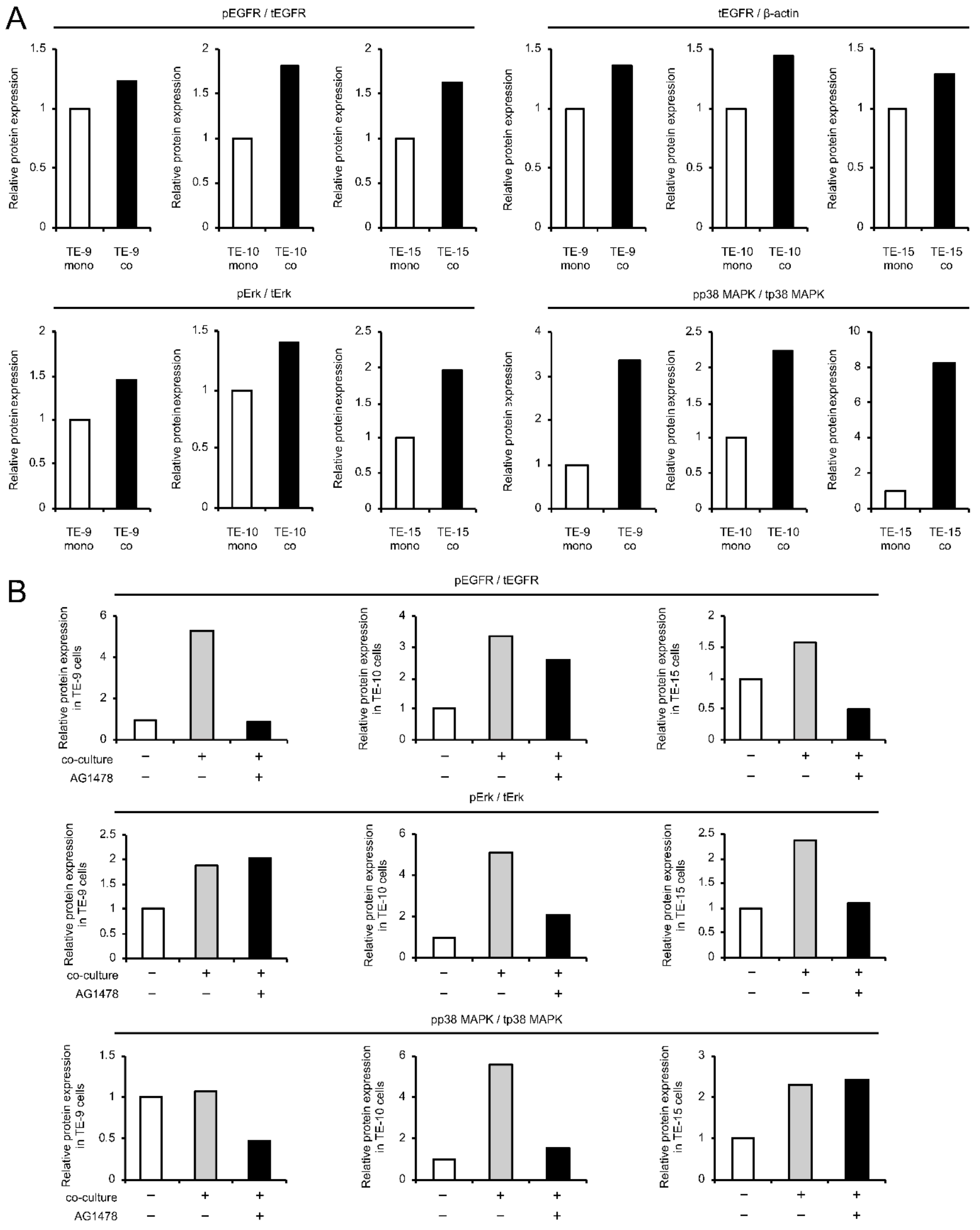

Figure S3. (A) Quantification of protein bands from Western blotting shown in Figure 2B. The protein expression levels of pEGFR (Tyr1068), EGFR, pErk, and pp38 MAPK were normalized to the tEGFR, β-actin, tErk, and tp38 MAPK, respectively. **(B)** Quantification of protein bands from Western blotting shown in Figure 2F. The protein expression levels of pEGFR (Tyr1068), pErk, and pp38 MAPK were normalized to the tEGFR, tErk, and tp38 MAPK, respectively.

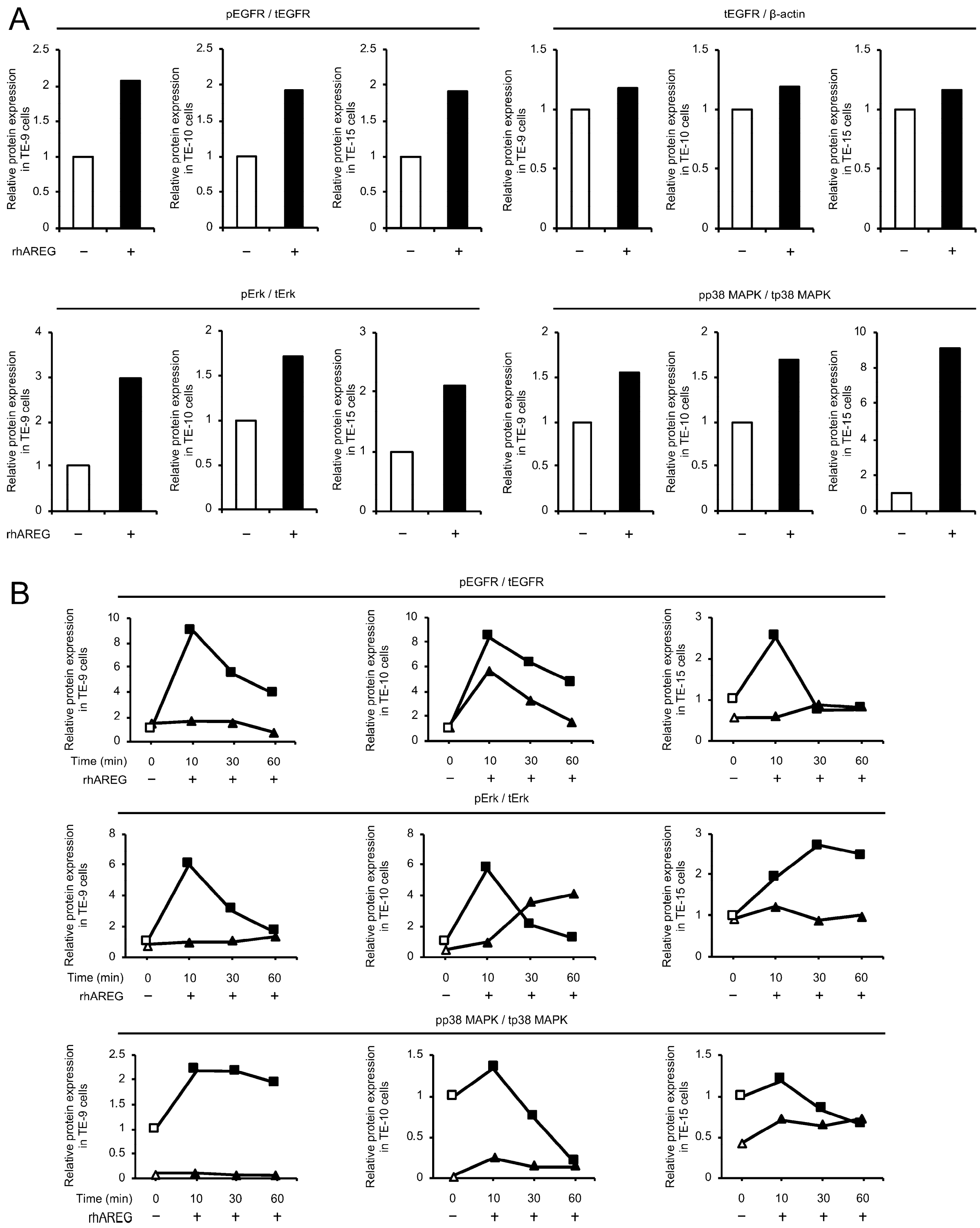

Figure S4. (A) Quantification of protein bands from Western blotting shown in Figure 3B. The protein expression levels of pEGFR (Tyr1068), EGFR, pErk, and pp38 MAPK were normalized to the tEGFR, β-actin, tErk, and tp38 MAPK, respectively. **(B)** Quantification of protein bands from Western blotting shown in Figure 3E. The protein expression levels of pEGFR (Tyr1068), pErk, and pp38 MAPK were normalized to the tEGFR, tErk, and tp38 MAPK, respectively. The data points depicted by squares represent the time course of expression levels in TE cells treated with DMSO, while the points shown as triangles represent the time course in TE cells treated with the EGFR inhibitor, AG1478.

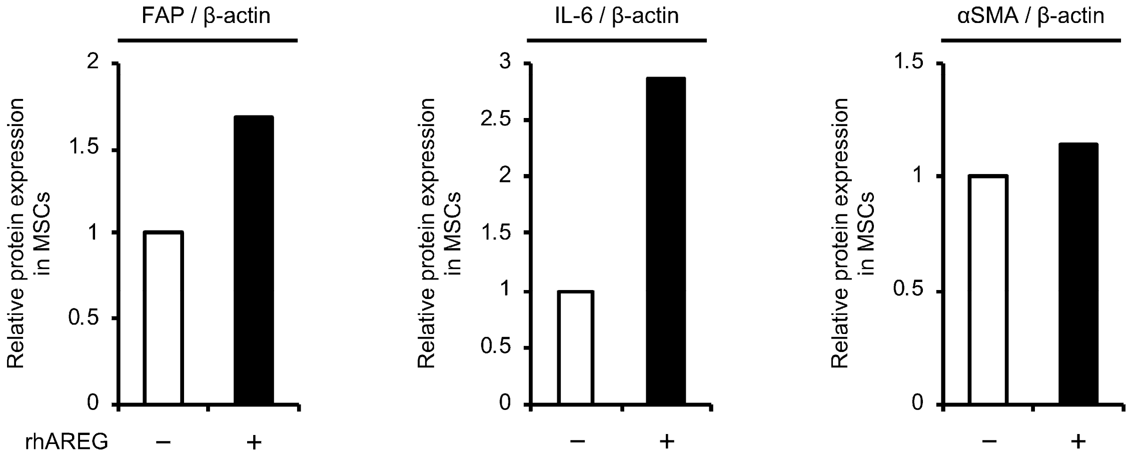

Figure S5. Quantification of protein bands from Western blotting shown in Figure 4B. The protein expression levels of FAP, IL-6, and αSMA were normalized to β-actin.

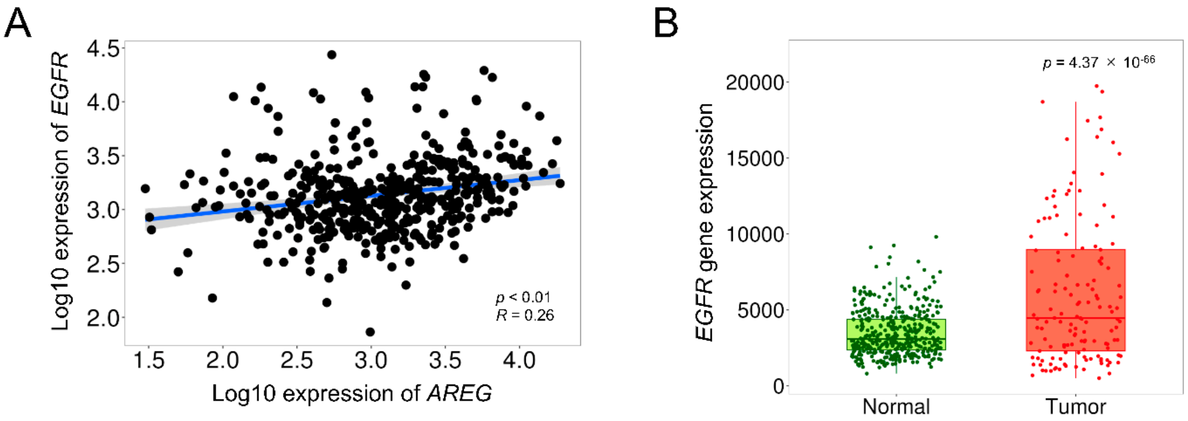

Figure S6. (A) Spearman correlation analysis of *AREG* and *EGFR* gene expression levels in ESCC tissues using the TNMplot database. **(B)** *EGFR* gene expression levels in normal and ESCC tissues using the TNMplot database.

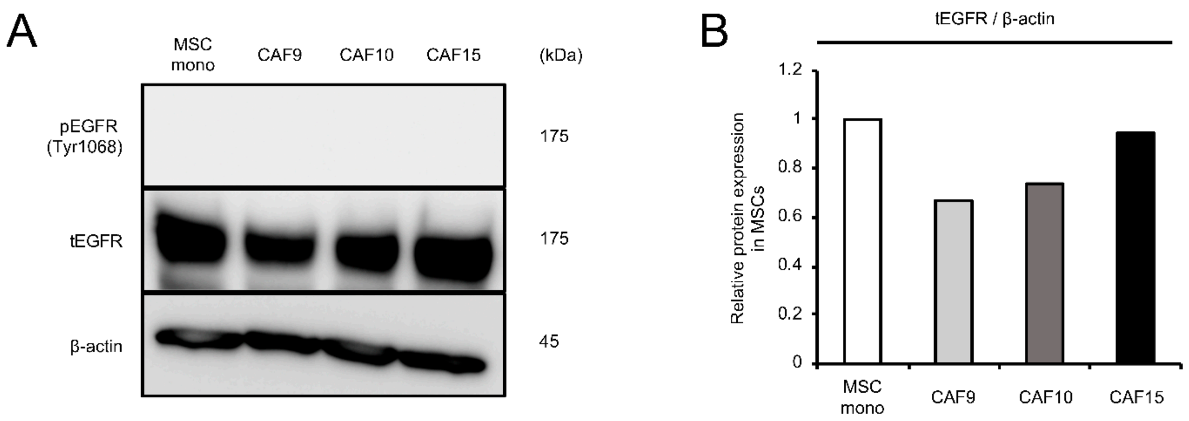

Figure S7. (A) The protein expression levels of EGFR and pEGFR (Tyr1068) in MSC mono and CAFs were compared using Western blotting. β-actin was used as a loading control. **(B)** Quantification of protein bands from Western blotting shown in Figure S7A. The protein expression levels of tEGFR were normalized to the β-actin.

A

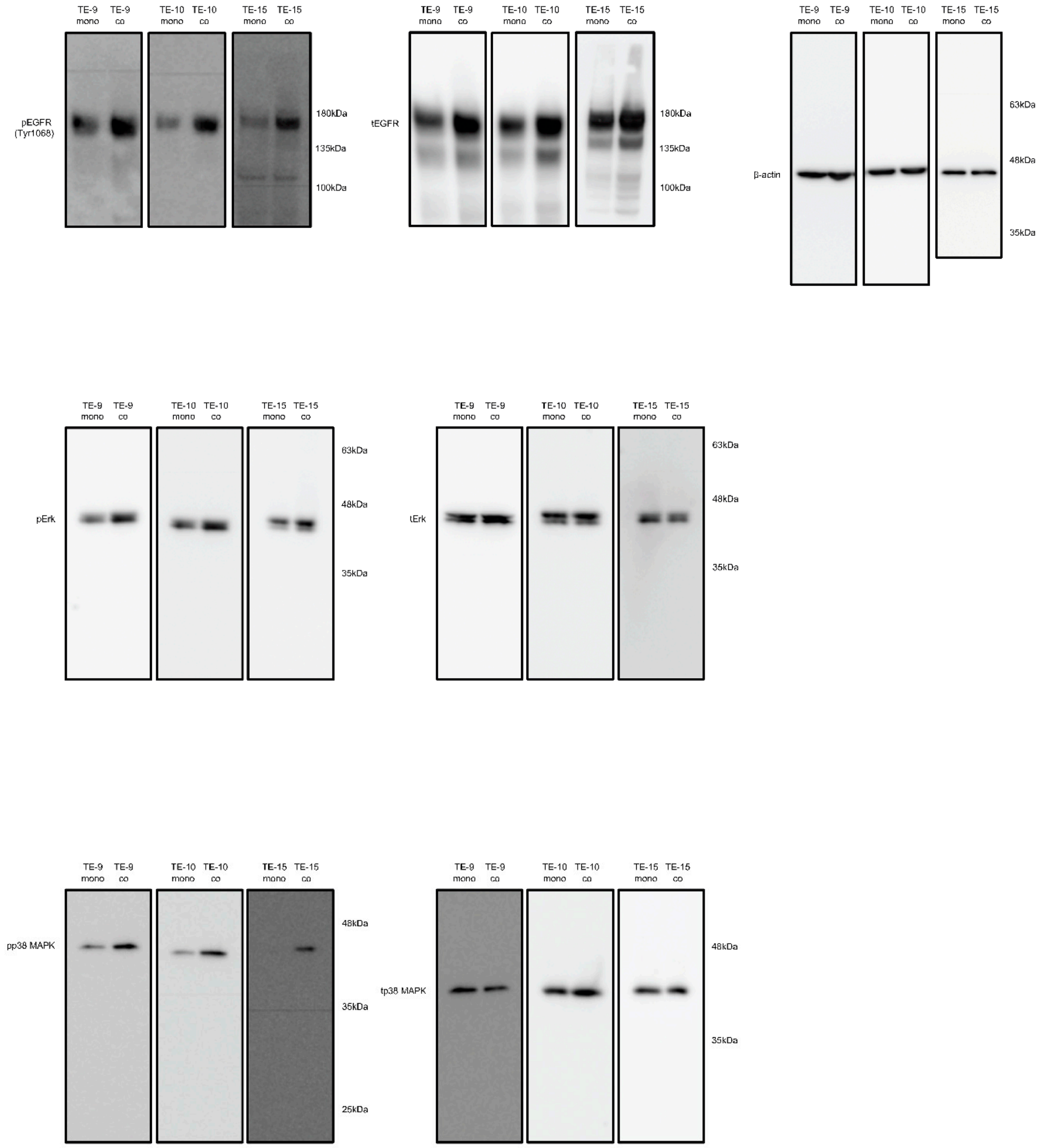
TE-9 mono
TE-9 co
TE-10 mono
TE-10 co
TE-15 mono
TE-15 co
pEGFR (Tyr1068)
tEGFR
β-actin
180kDa
135kDa
100kDa
63kDa
48kDa
35kDa
25kDa
pErk
tErk
pp38 MAPK
tp38 MAPK

B

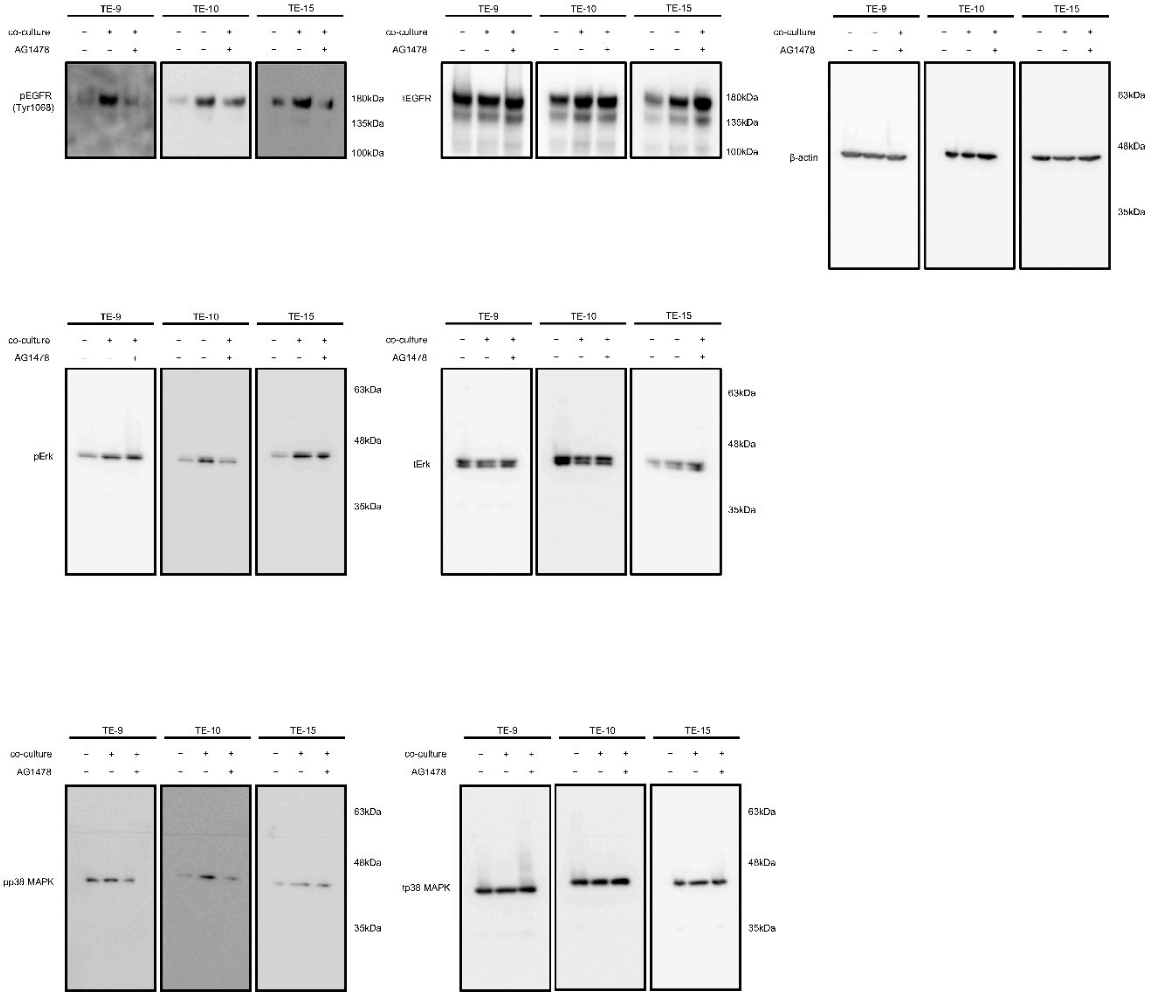
TE-9
TE-10
TE-15
co-culture
AG1478
pEGFR (Tyr1068)
180kDa
135kDa
100kDa
tEGFR
β-actin
63kDa
48kDa
35kDa
pErk
tErk
pp38 MAPK
tp38 MAPK

C

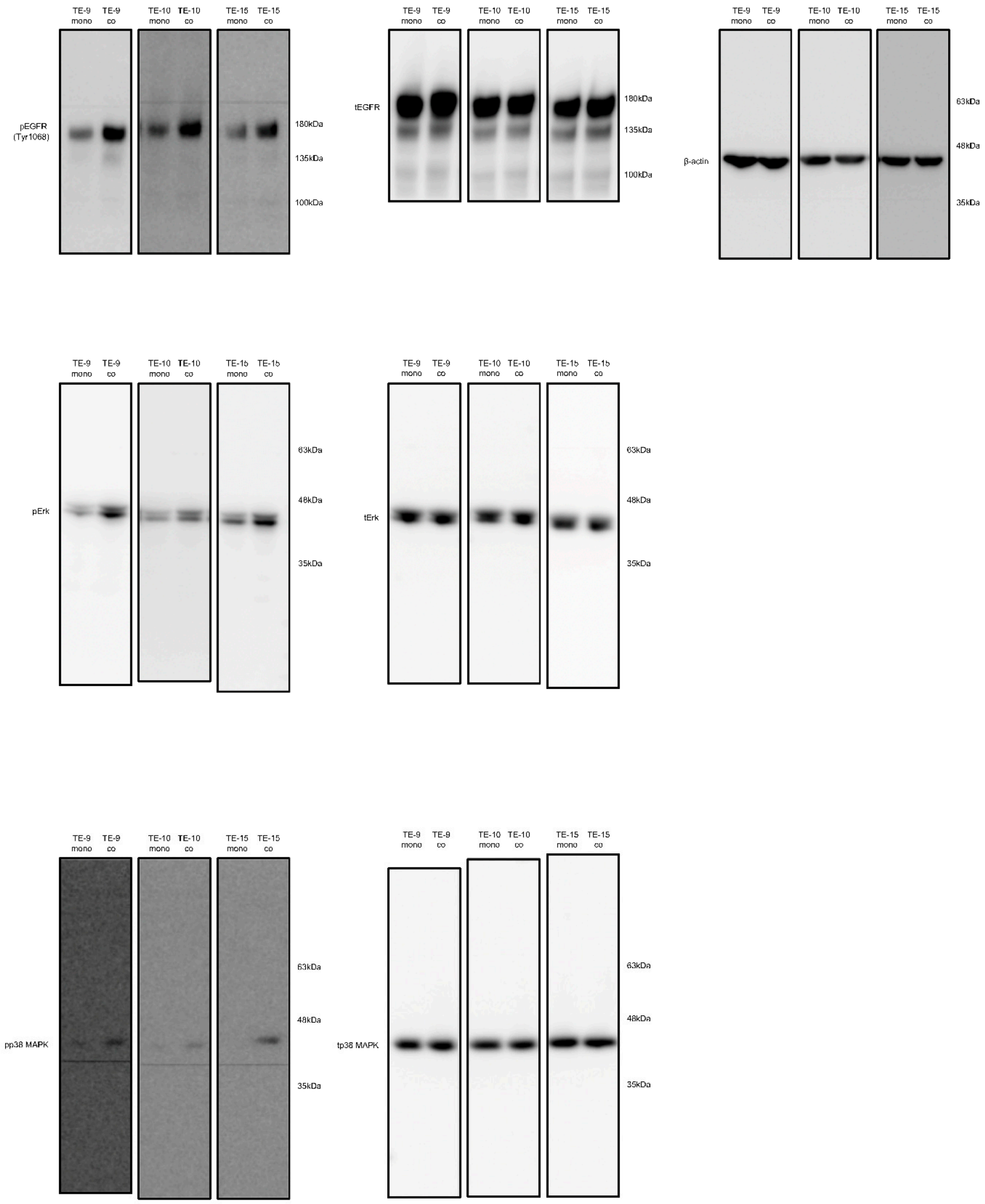
TE-9 mono
TE-9 co
TE-10 mono
TE-10 co
TE-15 mono
TE-15 co
pEGFR (Tyr1068)
180kDa
135kDa
100kDa
tEGFR
180kDa
135kDa
100kDa
β-actin
63kDa
48kDa
35kDa
pErk
63kDa
48kDa
35kDa
tErk
63kDa
48kDa
35kDa
pp38 MAPK
63kDa
48kDa
35kDa
tp38 MAPK
63kDa
48kDa
35kDa

D

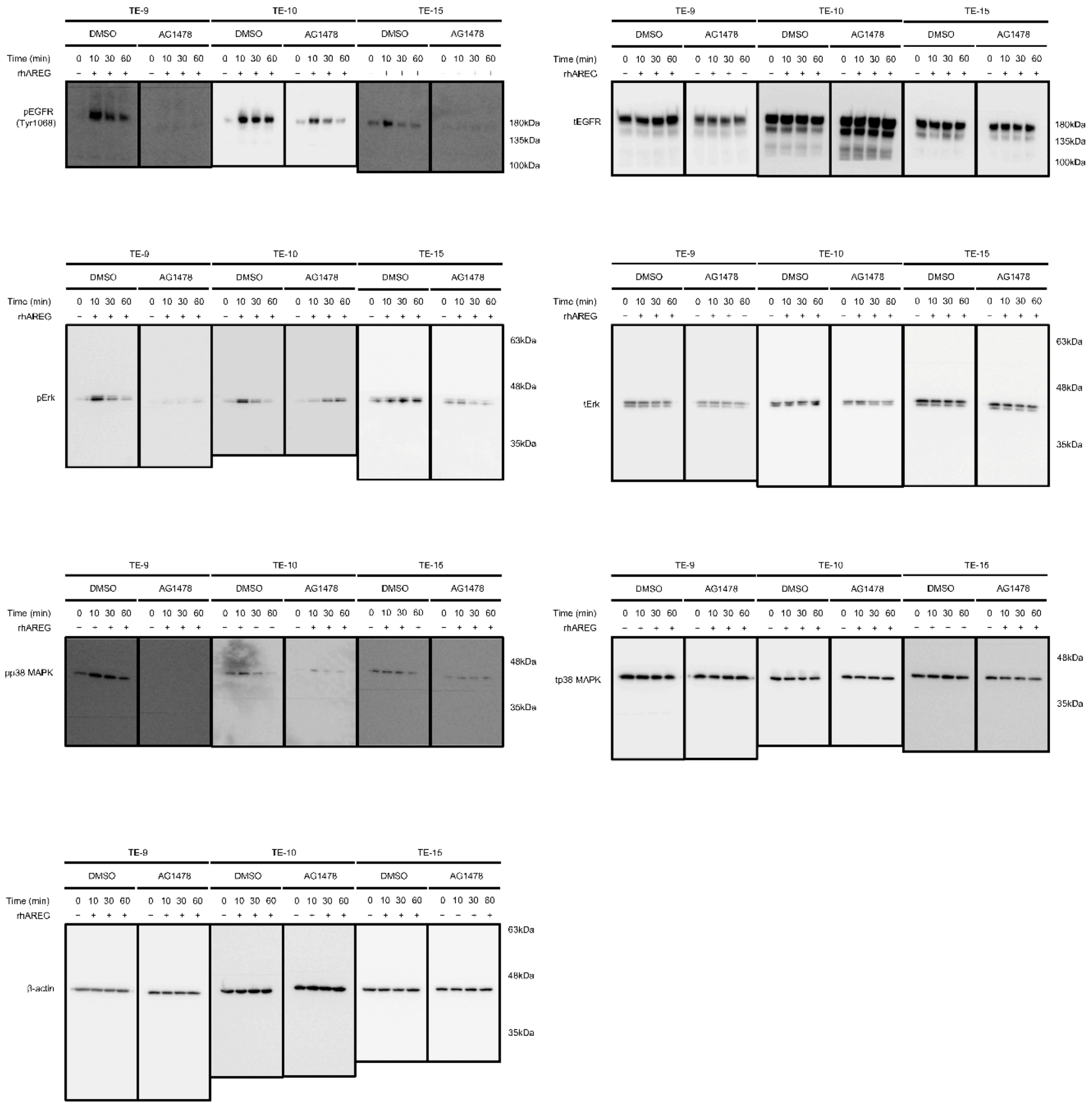
TE-9
TE-10
TE-15
DMSO
AG1478
Time (min)
0 10 30 60
rhAREG
pEGFR (Tyr1068)
tEGFR
180kDa
135kDa
100kDa
pErk
tErk
63kDa
48kDa
35kDa
pp38 MAPK
tp38 MAPK
β-actin

E

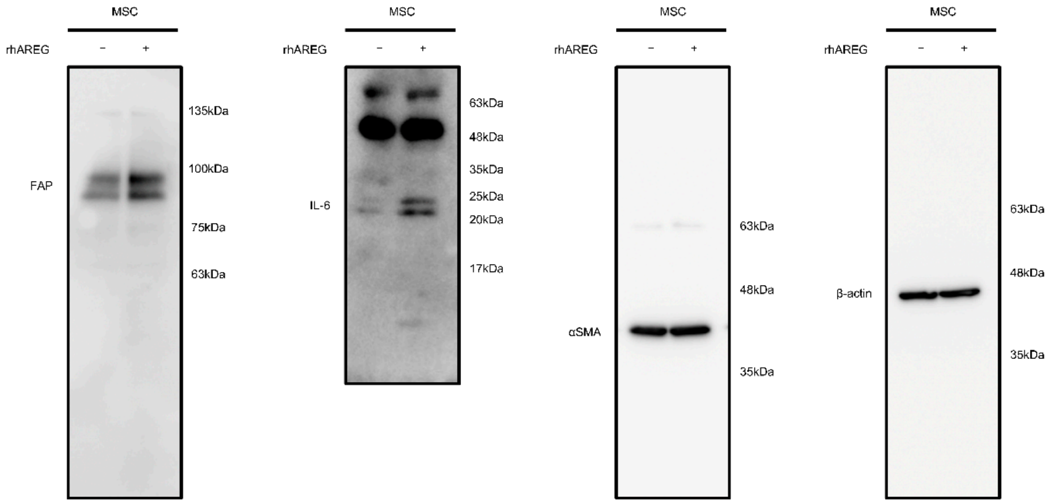
MSC
rhAREG
–
+
FAP
135kDa
100kDa
75kDa
63kDa
IL-6
63kDa
48kDa
35kDa
25kDa
20kDa
17kDa
αSMA
63kDa
48kDa
35kDa
β-actin
63kDa
48kDa
35kDa

F

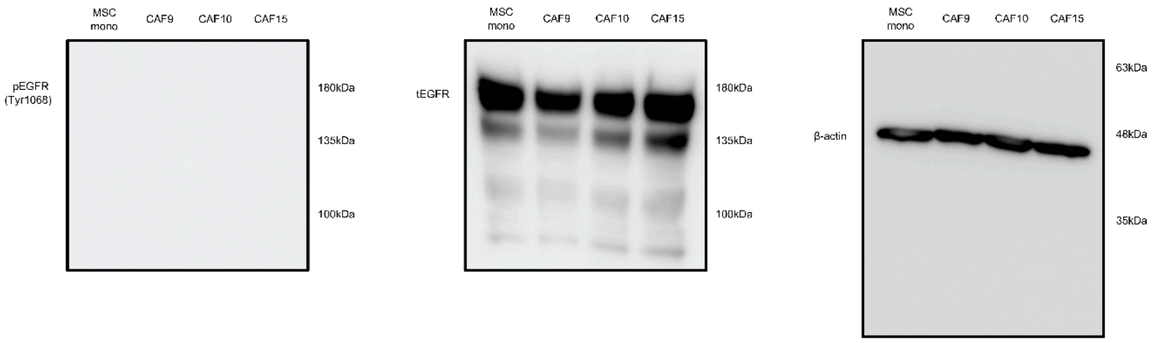

Figure S8. Raw Western blotting images corresponding to Figure 2B, 2F, 3B, 3E, 4B, and S7A are presented as Figure S8A–F, respectively. The protein markers are not depicted on these raw membranes.
